# Evaluation of Sample Size Influence on Chemical Characterization and In Vitro Antioxidant Properties of Flours Obtained from Mushroom Stems Coproducts

**DOI:** 10.3390/antiox13030349

**Published:** 2024-03-14

**Authors:** Patricia Bermúdez-Gómez, Juana Fernández-López, Margarita Pérez-Clavijo, Manuel Viuda-Martos

**Affiliations:** 1 Centro Tecnológico de Investigación del Champiñón en La Rioja (CTICH), Carretera Calahorra, KM 4, 26560 Autol, Spain; patricia.bermudez@goumh.umh.es (P.B.-G.); direccion@ctich.com (M.P.-C.); 2 IPOA Research Group, Institute for Agri-Food and Agri-Environmental Research and Innovation, Miguel Hernández University (CIAGRO-UMH), Ctra. Beniel km 3.2, 03312 Orihuela, Spain; mviuda@umh.es

**Keywords:** *Agaricus bisporus*, *Pleurotus ostreatus*, mushroom stem, mushroom co-products, sample size, antioxidant capacity, techno-functional properties, chemical characterization, bioactive compounds

## Abstract

The mushroom industry generates large amounts of stem co-product. This is generated after mushroom harvest; stems are attached to the growth substratum, and their only use has traditionally been as compost. In this study, we investigated extensively for the first time this co-product and the influence of sample size (L—>0.510 mm; LI—0.510–0.315 mm; SI—0.315–0.180 mm; S—<0.180 mm) on the characterization and antioxidant activity of flours obtained from stem co-products of *Agaricus bisporus* (ABSF) and *Pleurotus ostreatus* (POSF). ABSF was rich in protein (14 g/100 g), calcium (428.23–700.77 mg/100 g), and sorbitol (22.57–26.60 g/100 g), while POSF was rich in β-glucans (36.62–40.34 g/100 g) and linoleic acid (20.57–39.86 g/100 g of lipid). Both species were flush in amino acids and had an umami flavour. ABSF showed more elevated values for emulsifying activity than POSF. The S sizes were highlighted for their yield, hydration properties, and oil holding capacity. Furthermore, ABSF-S exhibited heightened antioxidant capacity in vitro, in consonance with the total phenolic compounds observed (0.91 mg/g). However, the antioxidant assays in POSF presented a positive correlation with β-glucan content. Our study suggests that these co-products could have several food-related applications, such as potential for use as an emulsifier, sweetener, or fortifier in the development of functional food, owing to their rich concentrations of fibre, protein, sorbitol, and β-glucans. Nevertheless, it is necessary to understand the interactions of the flours with the potential food matrix prior to proceeding further with food-related applications.

## 1. Introduction

Mushrooms are the fruiting bodies of *Basidiomycota* and some *Ascomycota* phylums from the *Dikarya* fungi subkingdom. These edible mushrooms are consumed worldwide, not only for their sensorial characteristics but also for their valuable nutrient composition; they are low in calories and fat, and rich in protein, dietary fibre, and vitamins [1,2,3,4]. Furthermore, in recent decades, potential biological activities such as anti-inflammatory, anticancer, hyperglycaemic, antibacterial, and antioxidant actions have been proven [5,6,7,8,9]. These potential health effects are attributed to the presence of secondary metabolites [7].

The valuable sensory and health characteristics of fruiting bodies have driven the worldwide production of cultivated edible mushrooms since the late 1990s. Overall, world mushroom production has been growing steadily since 1999, with production value reaching a total of US$ 46,608,283 in 2021 [4,10]. The most commonly cultivated species are *Agaricus bisporus*, *Lentinula edodes*, and *Pleurotus* [11]. Concretely, *A. bisporus* (AB) is the most extensively cultivated edible mushroom worldwide, with yields accounting for 30% of total edible fungi [5,12]. The second most popular species is the genus *Pleurotus*, within which more relevant is *Pleurotus ostreatus* (PO) [13,14]. The production of this genus on a global scale represents about 27% of total edible fungi [5]. During the commercialization of fruiting bodies, large quantities of co-products are generated, derived from two steps: cultivating the mushroom, and trimming. In the first step, spent mushroom substrate or mushroom compost is produced, and is the most abundant scrap. In the second step, mushroom stems and mushrooms whose size or shape do not meet commercial standards are discarded. Stem or stipe represents up to 20% of production volume [15,16,17]. This waste is usually managed via composting or incineration, both of which create an environmental problem due to unpleasant odour, attraction of disease-spreading insects, and air pollution [12,18,19]. The novelty of this study lies in the fact that it is the first complete study of the chemical composition, techno-functional, and physicochemical properties of stem waste, as well the first comprehensive investigation into the antioxidant activity of flours obtained from a co-product generated in the industrialization of mushrooms. In addition, it is the first study to analyse how particle size affects these properties. To our knowledge, in the scientific literature, it is possible to find studies related to the chemical composition of mushrooms, as well as several of their antioxidant or biological activities [1,2,3,4,5,6,7,8,9], but these studies refer to the fruiting body or the stems, and not to the co-product generated after the mushroom harvest, which is attached to the growth substratum. In order to accomplish the objectives of the 2030 Agenda for Sustainable Development, it is necessary to revalue agricultural waste with new alternative and profitable solutions.

Depending on the characteristics of the different co-products, they could be applied in the food, energy, pharmaceutical and cosmetic industries. *A. bisporus* stems (ABS) and *P. ostreatus* stems (POS) contain numerous bioactive compounds such as polysaccharides (β-D-glucans and chitin), minerals (zinc, iron, manganese, etc.), vitamin precursors (ergosterol), and amino acids (glutamic acid, alanine, valine, isoleucine, etc.) [1,3,4,16,20,21]. In recent years, mushrooms have been studied as a source for the extraction of compounds of interest such as ergosterol, peptides, phenolic compounds, glucans, and chitin [1,12,17,18,22,23]. Although numerous “green” extraction technologies have been developed in recent years, such as the use of pressurized liquids or supercritical fluids, these processes are expensive, still use cosolvents, and generate other residues. Considering the sustainable targets and the economics of the food industry, more environmentally friendly methodologies have been developed to revalue stems, such as incorporating them into food. To our knowledge, this methodology has been proven in noodles and bakery products, and in these related studies, differing functionality was seen between AB and PO [14,24]. In this context, ABS and POS could be used for generating intermediate food products by applying only physical processes.

The influence of sample size on components, antioxidant activity, and technological and physicochemical attributes has been investigated in several food matrices, but the extent of sample size influence in mushroom stem flours has yet to be established [25,26,27]. Therefore, the aim of this work was to study the influence of sample size and species type on the characterization and antioxidant activity of *A. bisporus* stem flour (ABSF) and *P. ostreatus* stem flour (POSF) in view of selecting, for each product, the most suitable food applications.

## 2. Materials and Methods

### 2.1. Materials

Sulfuric acid, sodium hydroxide, boric acid, hydrochloric acid, Kjeldahl tablets (catalyst containing 6.25% CuSO_4_—5H_2_O), acetone, ethanol, methanol, n-hexane, chloroform, glacial acetic acid, Folin–Ciocalteu reagent, anhydrous sodium carbonate, 2,2-azino-bis (3-ethylbenzothiazoline-6-sulfonic acid) (ABTS), and performic acid were provided by Panreac Química S.L.U. (Barcelona, Spain). Additionally, 3,4,5-trihydroxybenzoic (gallic) acid, 6-hydroxy-2,5,7,8-tetramethychroman-2-carboxylic acid (Trolox), 2,2-diphenyl-1-picrylhydrazyl (DPPH), hydrochloric acid, 2,4,6-tripyridyl-s-triazine solution (TPTZ), ferric (III) chloride, potassium persulfate, 2-(N-Morpholino) ethanesulfonic acid (MES), Tris(hydroxymethyl)aminomethane (TRIS), α-amylase solution, protease solution, amyloglucosidase solution, ninhydrin, and helium were provided by Sigma Aldrich (Steinheim, Germany). The amino acid standard mix used was composed of L-alanine (Ala), L-arginine (Arg), L-aspartic acid (Asp), L-cystine (Cys), L-glutamic acid (Glu), L-histidine hydrochloride monohydrate (His), glycine (Gly), L-isoleucine (Ile), L-leucine (Leu), L-lysine hydrochloride (Lys), L-methionine (Met), L-phenylalanine (Phe), L-proline (Pro), L-serine (Ser), L-threonine (Thr), L-tyrosine (Tyr), and L-valine (Val), all of which were provided by Sigma Aldrich (Steinheim, Germany). Distilled water was sourced from Millipore (Molsheim, France). Reference fat (BCR-164) and tritridecanoin were provided by Fedelco Inc. (Madrid, Spain). Organic acid (tartaric acid, lactic acid, acetic acid, isobutyric acid, citric acid), sugar (maltitol, sucrose, glucose, sorbitol, trehalose), and mineral (calcium (Ca), copper (Cu), iron (Fe), potassium (K), magnesium (Mg), manganese (Mn), sodium (Na), phosphorus (P), and zinc (Zn)) standards (purity ≥ 96%) were purchased from Supelco (Sigma-Aldrich, St. Louis, MO, USA).

### 2.2. Plant Material

**Figure 1:**
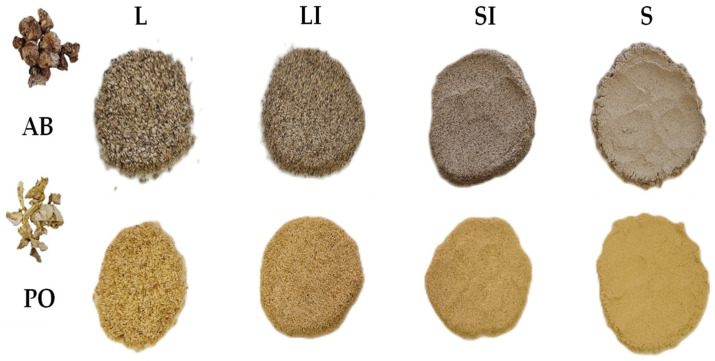
*A. bisporus* stems flour (ABSF) and *P. ostreatus* stems flour (POSF) at different sample sizes. L: >0.510 mm; LI: 0.510–0.315 mm; SI: 0.315–0.180 mm; S: <0.180 mm.

The *Agaricus bisporus* stems, of the strain Sylvan A15, were ceded from Cultivos Riojal (Autol, La Rioja, España) and were freshly cut after the fruiting body harvesting. The AB were grown on a substrate based mainly on wheat straw, chicken manure, and recirculated process water under temperatures between 15 and 19 °C and relative humidity ranging between 87 and 91% over a growing time of eight days. The sample was kept at 4 °C for no more than 5 days until it was cleaned with pressurized water and dried in a Master Jerky 550 dehydrator from Klarstein (Chal-Tec GmbH, Berlin, Germany) at 50 °C for 30 h. The *Pleurotus ostreatus* stems, of the strain Sylvan SPOPPO, were ceded from Micotec SA (Autol, La Rioja, España) and were freshly cut after the fruiting body harvesting. The PO were grown on a substrate based mainly on wheat straw under temperatures between 18 and 23 °C and relative humidity ranging between 90 and 95% over a growing time of 10 days. The sample was kept at 4 °C for no more than 5 days until it was dried in a dehydrator at 50 °C for 18 h. Dehydrated samples were grounded in ultracentrifugal mills (model ZM 200, Retsch™, Düsseldorf, Germany) at a sample size of 1 mm to obtain the flours. Both samples were sieved to produce different flours according to sample size. Each sample was separated into different fractions based on the sample size using three sieves with different apertures (0.510, 0.315, and 0.180 mm), and yield was calculated simultaneously. Thus, ABSF and POSF were divided into four sample size ranges: largest > 0.510 mm (ABSF-L and POSF-L), large-intermediate 0.510–0.315 mm (ABSF-LI and POSF-LI), small-intermediate 0.315–0.180 mm (ABSF-SI and POSF-SI), and smallest < 0.18 mm (ABSF-S and POSF-S). Figure 1 shows ABSF and POSF at different sample sizes. Samples were vacuum-packed and stored in darkness until analysis.

### 2.3. Proximate Composition

All determinations were performed in triplicate and expressed as g/100 g of flour. The proximate composition of mushrooms was expressed as a percentage of flour. Proximate compositions were estimated using methods based on the Association of Official Analytical Chemists [28]. Moisture contents were calculated using differences in weight after drying in a ED 400 stove (Binder, Tuttlingen, Germany) at 105 °C as compared to constant weight. Total ash was determined using a 10-PR/400 muffle (Hobersal, Barcelona, Spain) at 550 °C after 5 h, crude protein was determined using the kjeldahl method using a Kjeltec System 2200 nitrogen distiller (FOSS IBERIA, Barcelona, Spain) and a digestion block (FOSS IBERIA, Barcelona, Spain), and total dietary fiber (TDF), insoluble dietary fiber (IDF), and soluble dietary fibre (SDF) were estimated using methods based on the Association of Official Analytical Chemists [28]. Since mushroom contains nonprotein nitrogen, the factor used to calculate protein in this case was 4.38. Crude fats were extracted following the Folch method described by Eggers and Schwudke [29]; the samples were briefly hydrolysed with HCl (3 M) for 1 h at 80 °C in a WSB shaking water bath (Witeg, Wertheim, Germany), and finally fat was extracted via dilution (1:20, *v*/*v*) with chloroform:methanol (2:1, *v*/*v*). The dietary fibre was analysed according to the AOAC methods 991.43 [30], using α-amilasa, protease, and amyloglucosidase solutions to aid the digestion. Total carbohydrates were calculated by subtracting moisture, total fat, protein, and ash at 100%.

### 2.4. D-Glucans Profile

The beta-glucan content for each of the mushroom species was determined using a mushroom and yeast beta-glucan K-YBGL kit (Megazyme, Bray, Ireland). Extraction, laboratory analysis, and calculations were performed following the manufacturer’s instructions. The glucans were briefly hydrolysed and solubilised by adding sulfuric acid (12 M) under a combination of high and low temperatures. Then, the total glucan content was determined spectrophotometrically using a GENESYS™ 10S UV-Vis spectrophotometer (ThermoScientific, Madrid, Spain) with a wavelength of 510 nm. D-glucose at 1 mg/mL was used as the calibration compound. A similar procedure was carried out to obtain the α-glucan content, but NaOH (1.7 M) was used in place of sulfuric acid. Finally, β-glucan content was calculated through finding the difference between total glucan content and α-glucan content. All analyses were performed in triplicate, and results were expressed as g/100 g of flour.

### 2.5. Physicochemical Analysis

The pH was measured in 10% (*w*/*v*) aqueous solutions of the samples in a pH meter GLP 21 (Crison Instrument S.A., Barcelona, Spain). Water activity (Aw) was determined using a Novasina Thermoconstanter Sprint TH-500 (Novasina, Pfäffikon, Switzerland) at 25 °C. Colour was measured with a BGD 551 colorimeter (Biuged Precise Instruments Co., Guangzhou, China) with illuminant D_65_, observer 8°, SCI mode, an 8 mm aperture for illumination, and a 4 mm aperture for measurement, based in the CIEL*a*b* colour space. The following colour coordinates were determined: lightness (L*), redness (a* ± red-green), and yellowness (b* ± yellow-blue). From these coordinates, hue (h*) and chroma (C*) were calculated using Equations (1) and (2), respectively. Color difference (ΔE) was calculated based on the largest size using Equation (3).

(1) $$h^{*}={tan}^{-1}\frac{b^{*}}{a^{*}}$$

(2) $$C^{*}={\left(a^{*2}+b^{*2}\right)}^{\frac{1}{2}}$$

(3) $$\Delta E=\sqrt{{{(∆L}^{*})}^{2}+{{(∆a}^{*})}^{2}{{(∆b}^{*})}^{2}}$$

### 2.6. Techno-Functional Properties

Water and oil holding capacity (WHC and OHC, respectively) were determined following the methodology described by Chau et al. [31]. For WHC, 10 mL of ultrapure water was added into a centrifuge tube containing 300 mg of ABSF or POSF sample. Subsequently, the centrifuge tubes were stored at 25 °C for 18 h. After being centrifuged (1500× *g*, 20 min), the supernatant was discarded and the pellet was weighed. The WHC of each sample was expressed as the weight of water held by 1 g of the corresponding sample. OHC was determined following the same methodolgy, with water being replaced by oil. The results were expressed as the weight of oil held by 1 g of the corresponding sample. Beuchat’s method was employed for the determination of water absorption capacity (WAC) [32]. The results were reported as g of water or oil held by g of sample (g/g). Emulsifying activity (EA) assessment was carried out following the methods described by Yasumatsu et al. [33]. In brief, 1 g of each of the ABSF and POSF samples were weighed into separate 150 mL flasks. Then, 50 mL of ultrapure water was added and mixture was homogenized at 8000 rpm for 2 min (Ultra-Turrax T25 BASIC, IKA-Werke GmbH and Co. KG, Staufen, Germany) to obtain an aqueous fiber suspension. Fifty milliliters of sunflower oil was then added into the slurry, and this was further homogenized at 8000 rpm for 1 min. An aliquot (25 mL) of the emulsion formed was transferred into graduated centrifuged tubes (1500 rpm, 25 °C, 5 min). The EA was calculated (Equation (4)) from the ratio of the depth of the emulsified layer to the depth of the total volume of content inside the centrifuge tube (as a percentage).

(4) $$EA=\frac{Emulsified\;layer\;(mL)}{Total\;sample\;volume\;(mL)}\times 100$$

Swelling capacity (SWC) was measured following the method described by Robertson et al. [34]. In brief, ABSF and POSF samples (500 mg) were weighed in 10 mL measuring tubes, and 5 mL of ultrapure water was added to each. Then, the mixtures were stirred gently to eliminate trapped air bubbles, and left on a level surface at room temperature for 24 h to allow the samples to settle. The volume (mL) occupied by the hydrated samples was measured, and the results were expressed as mL water per g of sample (mL/g). Finally, gelation properties were analysed following the method described by Chau et al., and the results were expressed as the least gelation concentration (LGC) [35].

### 2.7. Amino Acids Profile Analysis

The total protein amino acids were analyzed according to the method established by the European Commission and following the method described by Mattila et al. [36]. Amino acids were determined by reaction with ninhydrin after acid hydrolysis (6 M HCl, 110 °C, 24 h) using a Biochrom 20 amino acid analyzer (Pharmacia Biotech, Cambridge, UK) equipped with a 90 × 4.6 mm PEEK sodium pre-wash column and 250 × 4.6 mm Bio PEEK sodium high performance column (Pharmacia Biotech, Cambridge, UK). The sulfur-containing amino acids cysteine and methionine were oxidized with performic acid (0 °C, 16 h) to cysteic acid and methionine sulfone prior to acid hydrolysis and calculated as cystine and methionine, respectively. The hydrolysis acid contained 1 mg of phenol per mL of acid to protect labile amino acids, especially tyrosine and phenylalanine, both of which were determined in hydrolysates of unoxidized samples. Other amino acids were determined as mean values of the oxidized and unoxidized samples. Tryptophan is all but destroyed by acid hydrolysis, and was not determined in this study.

All analyses were performed in triplicate, and results were expressed as g/100 g of flour. The amino acid scoring pattern (AASP) was extracted from a report about dietary protein quality evaluation provided via an FAO expert consultation [37]. The amino acid score (AAS %) was calculated by dividing the amino acid content in 100 g of protein by the AASP.

### 2.8. Minerals Profile Analysis

The mineral content was determined using inductively coupled plasma-mass spectrometry (ICP-MS, Shimadzu MS-2030, Shimadzu, Kioto, Japan), following the method described by Muñoz-Bas et al. [38]. The standard compounds were diluted and utilized to calibrate the ICP-MS for mineral analysis in ABS and POS samples. ICP-MS operated under the following conditions: carrier gas 0.70 L/min; plasma gas 9.0 L/min; auxiliary gas 1.10 L/min; radio frequency 1.2 kW; and energy filter 7.0 V. All analyses were performed in triplicate, and results were expressed as mg/100 g of flour.

### 2.9. Sugars and Organic Acids Profile Analysis

For the extraction of sugars and organic acids from flour samples, 2 g of each flour was mixed with 50 mL of ultrapure water and stirred at room temperature for 24 h. Then, these solutions were homogenized at 20,000 rpm for 2 min (Ultra-Turrax T25 BASIC, IKA-Werke GmbH and Co. KG, Staufen, Germany) and heated at 80 °C for 1 h under constant stirring. Following centrifugation (5000 rpm for 10 min at 4 °C), the supernatant was filtered through a 0.45 µm filter. Finally, organic acids and sugars were quantified via HPLC analysis (Hewlett-Packard 1100 series model, Woldbronn, Germany), following the procedure described by Muñoz-Bas et al. [38]. The samples were injected into a Supelco column (Supelcogel TM C-610H column 300 mm × 78 mm) using an elution buffer, ortho-phosphoricacid, in water (0.1% *v*/*v*), with an isocratic flow rate of 0.5 mL/min. For organic acid determination, the absorbance was measured at 210 nm using a diode-array detector (DAD G-1315A, Hewlett-Packard, Woldbronn, Germany), while sugar determination was carried out by means of a refractive index detector (RID G1362A, Hewlett-Packard, Woldbronn, Germany). All analyses were performed in triplicate, and results were expressed as g/100 g of flour.

### 2.10. Fatty Acids Profile

Mushroom stem oil was extracted from 25 g of samples using n-hexane via ultrasonic extraction at room temperature for 30 min (solid-liquid ratio of 1:4, *w*/*v*). After the extraction, the liquid was collected in a flask and the solvent was removed through a rotary vacuum evaporator. Fatty acid composition identification was accomplished via transesterification of fats with methanol, producing fatty acids methyl esters (FAME), as described by Golay et al. [39]. Gas chromatography (GC) analysis was carried out using an autosystem chromatographer (Perkin Elmer, Beaconsfield, UK) equipped with a VF–23 ms fused silica capillary column (30 × 0.25 mm × 0.25 μm film thickness) from Varian Inc. (Middelburg, The Netherlands) and a flame ionization detector (FID). The column was maintained at 60 °C for 1 min after injection; then, the temperature was set at 10 °C/min until it reached 130 °C, then set at 3 °C/min until 170 °C, and finally at 10 °C/min until 230 °C, at which point the temperature was held for 5 min. Helium was used as a carrier gas with a column inlet pressure set at 20 psi and a split ratio of 1:20. The injection volume was 0.5 μL. The total race time was 32 min. The injector and detector temperatures were set at 250 °C and 270 °C, respectively, as described by Pellegrini et al. [40]. All analyses were performed in triplicate, and results were expressed as percentage (%) of total fatty acids.

### 2.11. Antioxidant Compounds and Capacity

#### 2.11.1. Extraction Method

The methodology described by Delgado-Ospina et al. was followed, with some modifications, to extract polyphenols and antioxidant molecules in ABSF and POSF [41]. In brief, 3.0 g of each sample was mixed separately with 10 mL of methanol:water mixture (80:20, *v*/*v*), vortexed in a RSLAB-6 PRO vortex (RSLab, Parana, Argentina) at 20,000 rpm for 1 min, and then sonicated in a Ultrasons-HD (Selecta JP, Barcelona, Spain) for 10 min at 35 °C. After centrifugation (5000 rpm for 10 min at 4 °C), the supernatants were collected. The process was repeated twice with the successive pellet. Finally, the pellet was mixed with 10 mL of acetone:water mixture (70:30, *v*/*v*), and the same procedure was repeated. The supernatants were combined and evaporated to dryness using a SyncorePlus instrument (Büchi Labortechnik AG, Flawil, Switzerland). The yield of the extracts obtained was 47% for ABSF-S, and 42% for POSF-S. The other samples produced a yield of between 19 and 30%. The ABSF and POSF drying extracts were resuspended in methanol:water mixture (80:20, *v*/*v*) before proceeding to the following analysis.

#### 2.11.2. Assessment of the Total Phenolic Content

Total phenolic content (TPC) was determined by the Folin-Ciocalteu method as described by Liu et al. [42]. The results were expressed as mg of gallic acid equivalents per g of flour (mg GAE/g of flour).

#### 2.11.3. Evaluation of the In Vitro Antioxidant Capacity

The antioxidant activity of the extracts was determined by the ABTS, DPPH, and ferric-reducing antioxidant power (FRAP) methods. Results were expressed as Trolox equivalent antioxidant capacity (TEAC) or µmol of Trolox equivalent/g flour (µmol/g), calculated based on a calibration curve of Trolox. A microplate spectrophotometer, Multiskan Skyhigh (Thermo Scientific, Waltham, MA, USA), was used in all methods. The ABTS assay was performed as described by Re et al. [43]. In brief, the solution of ABTS cation chromophore was prepared by mixing ABTS (7 mM) with potassium persulfate solution (139.8 mM). The mixture was kept in darkness at 25 °C for 16 h and adjusted to absorbance values of 0.70 ± 0.2 at 734 nm via dilution with absolute methanol. The assay was conducted in a 96-well plate by mixing 10 µL of each extract sample and 290 µL of the ABTS solution. Absorbance values were measured on a spectrophotometer at 734 nm. DPPH scavenging ability was determined according to the methodology described by Brand Williams et al. [44]. Initially, 100 µL of different concentrations of each extract sample was mixed with 150 µL of the 7.6 µM DPPH solution in methanol. The mixtures were incubated at room temperature for 30 min in darkness. Absorbance values were measured on a spectrophotometer at 517 nm. Finally, FRAP was analysed using the potassium ferricyanide-ferric chloride method [45]. Initially, the FRAP solution was prepared by mixing TPTZ (2,4,6-Tri(2-pyridyl)-s-triazine) (10 mmol/L), HCl (40 mmol/L), acetate buffer (300 mmol/L, pH 3.6), and FeCl_3_·H_2_O (20 mmol/L). Then, an aliquot of 10 µL of the mushroom extract was mixed with 240 µL of the FRAP solution in a 96-well plate and incubated for 10 min at 37 °C in darkness. Absorbance values were measured on a spectrophotometer at 593 nm.

### 2.12. Statistical Analysis

For each experiment, three independent samples were examined, with three replications per sample. Data obtained for all the determinations were analysed by means of a two-way ANOVA test with two factors: species and sample size. Tukey’s post hoc test was applied for comparisons of means; differences were considered significant at *p* < 0.05. Before Tukey’s post hoc test, the data were normalized when the variances of the samples analysed were different. After Tukey’s post hoc test, numerical intervals were marked to select colours in a heat map according to significant differences. Analysis was performed to determine relationships between sample size and all results of the mentioned examinations using Pearson correlation analysis. Statistical analyses were carried out using the statistical package SPSS 19.0 (SPSS Inc., Chicago, IL, USA).

## 3. Results and Discussion

### 3.1. Proximate Composition

The proximate composition of the flours was discretely influenced by sample size and species (*p* < 0.05). According to the results (Table 1), the main component in both mushroom flours was total carbohydrates (*p* < 0.05), as previously reported [1]. The high total carbohydrate content of both ABSF and POSF (values between 68.27 and 70.63 and 72.70 and 74.48 g/100 g, respectively) was due to the structure of the fungal cell wall and the role of the stem. The stem is the morphological part of the mushroom that manages the functions of transport and support, and thus stems contain well-developed transport and mechanical tissue structures made up of polysaccharides [46,47]. The moisture obtained in all samples was lower than the 12% recommended for the stability and safety of co-products [48]. The protein content of ABSF was about 14 g/100 g in all sample sizes. This value was lower than the 17.22 g/100 g previously reported by Umaña et al. [1] and the 15.30 g/100 g described by Cherno et al. [47]. Considering the results acquired, ABSF could be a source of protein according to the nutritional claim determined by the Codex Alimentarius [49]. POSF-S showed the highest protein content (9.42 g/100 g) of the POSF samples, which was lower than that of ABSF (*p* < 0.05), but similar to that described for PO stems (10 g/100 g) by Cherno et al. [47]. The lipid content of both ABSF-L and POSF-L was in accordance with the previously reported data for the stems of both species (1.54–2.00 g/100 g), but in the rest of the samples, it was higher than indicated by previous studies, especially in the S sizes. Therefore, it could be interesting to further study the lipid profile of the flours [1,47]. Differences in proximal composition could be due to the involvement of many factors, such as maturation stage, strain, location, and substrate [5]. Based on this result, ABSF could be incorporated into foods to increase their protein content.

According to previous studies, mushrooms are mainly comprised of non-digestible carbohydrates due to the structure of their cell walls [50]. Consequently, mushrooms are often considered a main source of dietary fibre. Table 1 shows the dietary fibre results for ABSF and POSF. POSF, at all sample sizes, denoted a higher TDF content than ABSF (*p* < 0.05). This amount was also higher than those observed in other high-fibre co-products, such as cacao pod husk (35.3–37 g/100 g dw) and cacao shell (48.1 g/100 g dw) [41]. All samples analysed exhibited a higher IDF than SDF content (*p* < 0.05). IDF represents more than 90% of TDF in both ABSF and POSF. This fact has been reported by other authors previously [51,52]. It is generally accepted that an SDF/IDF ratio close to 1:2 in dietary fibre sources could contribute not only to the enhancement of functionality and health benefits, but also to improving sensorial attributes [53,54]. It is important to highlight the effect of sample size on the SDF/IDF ratio. In the present study, SDF increased with a decrease in sample size due to the negative correlation presented between SDF content and the sample size in both ABSF and POSF (r = −0.97 and r = −0.73, respectively), while between IDF content and sample size, there was a positive correlation (r = 0.91 for ABSF, and r = 0.98 for POSF). Although mushroom flours showed higher SDF/IDF ratios than 1:2, the samples closest to this ratio based on the present correlation were ABSF-S (1:10) and POSF-S (1:9.59). In general terms, there is not much difference in the chemical composition depending on the particle size, so it would be necessary to use other criteria such as techno-functional, physicochemical, or antioxidant properties to elucidate which type of flour is most convenient to use as an ingredient in the food industry when new foods are going to be developed.

### 3.2. Sample Size Distribution Yield

**Figure 2:**
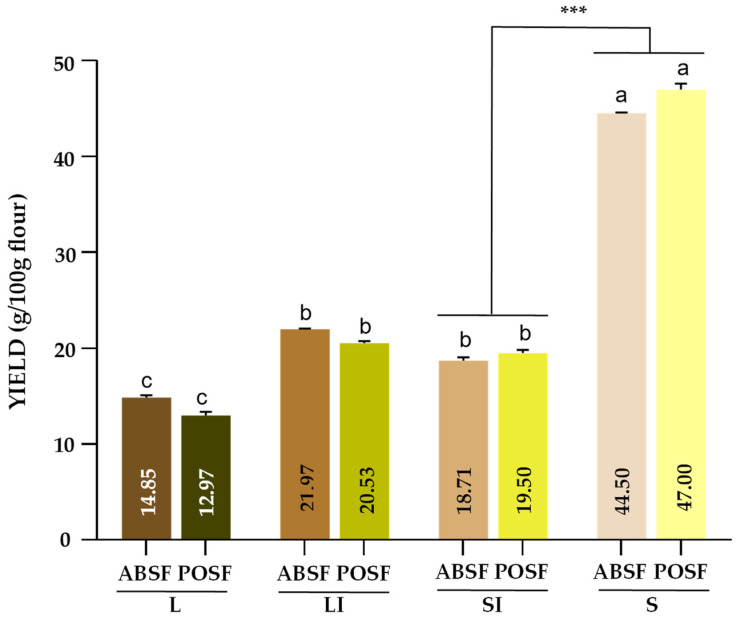
Sample size distribution yield of *A. bisporus* stems flour (ABSF) and *P. ostreatus* stems flour (POSF). Results are reported as mean ± SD (n = 3). Different letters (a–c) are significantly different when subjected to Tukey’s test (*p* < 0.05). *** *p* < 0.05. L: >0.510 mm; LI: 0.510–0.315 mm; SI: 0.315–0.180 mm; S: <0.180 mm. Results are expressed as g/100 g of flour.

In order to compare the economic value of the flours at different sample sizes, the obtained yields were calculated (Figure 2). The distribution of the flour into sample sizes was not influenced by the species type (*p* > 0.05). The highest yields observed were in the smallest sizes of ABSF and POSF, which were both near to 50%. These results were in consonance with those previously reported by Aguiló-Aguayo et al. [52], who examined mushroom by-product powders with sample sizes ranging from 90 to 425 µm. In this context, samples of sizes lower than 180 µm could be more suitable for incorporating as ingredients in the food industry for economic reasons as well as for industrial handling. However, their use in a pilot plant should be tested prior to commercialisation.

### 3.3. D-Glucans Profile

Mushroom dietary fibre is made up of some compounds of industrial interest, such as chitin and D-glucans [4]. The total α- and β-glucan content in ABSF and POSF at different sample sizes is summarised in Table 1. POSF showed the highest total glucan and β-glucan content (*p* < 0.05). All samples analysed exhibited higher β-glucan than α-glucan content (*p* < 0.05), except ABSF-S. This is in agreement with the observed decrease (*p* < 0.05) in β-glucans between small-intermediate (13.85 g/100 g) and the smallest (9.51 g/100 g) samples. This great difference could be due to the hypothesis described by Aguiló-Aguayo et al. [52]. According to this hypothesis, the finer the sample size, the higher the diffusion resistance and the lower the hydrolysis. Total D-glucan and β-glucan content analysed for ABSF was higher than that previously reported for AB stems, while the level of α-glucan content was in agreement with the findings reported by Umaña et al. [1,52]. The values obtained for α-glucans in POSF ranged from 10.45 to 12.72 g/100 g, which was higher than the amount indicated for PO stems by Synytsya et al. [51]. By contrast, observed β-glucans in POSF (values between 36.62 and 40.34 g/100 g) were within the range obtained previously for PO stems of different strains [51]. The variance observed could be due to the strain used. With regards to sample size effect, the highest β-glucan contents were observed in the smallest sample size of POSF. Based on this determination, all ABSF and POSF flours, but especially POSF-S could be used in the development of functional foods due to the potential biological benefits of the contained β-glucans, including antioxidant, antitumor, and immunomodulating activities [55]. However, the effect of digestion and the interaction with the food matrix must be studied before claiming this definitively.

### 3.4. Physicochemical Analysis

All physicochemical parameters studied in ABSF and POSF (Table 2), except pH, were influenced by sample size and species analysed (*p* < 0.05).

#### 3.4.1. Water Activity and pH

In the case of Aw, all of the samples analysed had values ranging between 0.38 and 0.50. ABSF showed lower values (*p* < 0.05) than POSF. These Aw values were within the range reported for other food co-products like pear, date fruits, and persimmon [27]. Due to their Aw values, both ABSF and POSF seem to have a low risk for deterioration as a result of microorganisms and enzymatic or non-enzymatic reactions. On the other hand, given their pH values (6.07–6.21), all samples seem to have a high risk, and this should be considered.

#### 3.4.2. Colour Parameters

In reference to colour parameters, the drying process changed the colour of samples from white in appearance to dark, in concurrence with what has been previously reported [56]. In addition, it is important to highlight that there are differences between fruiting bodies and stems. The results in this work (Table 2) were similar in yellowness (b*) but higher in lightness (L*) and redness (a*) than those previously reported by Zawadzka et al. for PO stems [21]. There is no previous reference available regarding the colour change observed in drying AB stems. In both ABSF and POSF, L* and hue (h*) values decreased with increasing sample size (*p* < 0.05), with the maximum L* and h* values belonging to the S size. The L* parameter difference could be explained by the increase in light reflection with decreasing sample size [27]. For the same sample size, POSF showed higher (*p* < 0.05) L* and h* values than ABSF. Hue values in all of the samples were in the range of orange h* (60–90°), and this angle became closer to orange-yellowish with decreasing sample size (*p* < 0.05) [57]. Values of a* decreased with decreasing sample size from L to S (*p* < 0.05) in POSF, with a Pearson correlation value of r = 0.89. The colour parameters corresponding with b* and chroma (C*) in POSF (values between 27.68 and 29.62 and 27.71 and 29.69, respectively) were higher than those in ABSF (*p* < 0.05), which ranged from 14.12 to 15.06 in b*, and 14.82 to 15.69 in C*. These significant differences in yellowness, chroma, and hue values related to species could be due to the higher glucan or carotenoid content of POSF, which are pigments with orange-yellow colours. On the other hand, the loss of redness observed in POSF could be due to the loss of another pigment during milling. Important differences analysed related to the previously mentioned parameters were in consonace with the ΔE values. The color differences specifically were appreciable to the human eye at all particle sizes (ΔE* > 4), except in the large-intermediate size of ABSF. In order to obtain a suitable ingredient, changes in colour parameters must be taken into account before addition to food, since the colour is one of the most important sensorial attributes for consumer acceptance [58,59].

### 3.5. Techno-Functional Properties

Table 3 shows the results obtained for the techno-functional properties of ABSF and POSF at different sample sizes (*p* < 0.05).

#### 3.5.1. Water Holding, Water Absorption, and Swelling Capacity

All studied hydration properties (WHC, WAC, and SWC) were discretely influenced by sample size in ABSF (*p* < 0.05), whereas only SWC and WAC were affected in POSF (*p* < 0.05). Some authors have previously suggested a decrease in WHC coincides with a loss of cell wall integrity, in addition to a positive correlation between this hydration property and dietary fibre content; the results obtained in this work corroborated this correlation in ABSF (r = 0.81) [60]. The highest WHC was observed in ABSF-LI (6.84 g/g), in consonance with TDF (Table 1). On the other hand, WHC was higher in ABSF (*p* < 0.05) than in POSF (values from 5.03 to 6.84 and 3.64 to 3.93 g/g, respectively), contrary to TDF (Table 1); this could be due to a higher cellulose content [60,61]. In the case of WAC, the highest values were observed in ABSF-S and POSF-S (5.27 and 4.10 g/g, respectively), and the same was observed for SWC in ABSF-S (15.39 mL/g). There was no difference (*p* > 0.05) between the SWC of ABSF and POSF at any of the sample sizes, except at the largest size. There was a negative correlation between WAC and IDF content in both ABSF and POSF (r = −0.99 and r = −0.98, respectively) and a positive correlation with SDF in both samples (r = 0.99 and r = 0.92, respectively). In this context, the difference found could be due not only to a higher contact surface area, but also to the IDF: SDF ratio, which could mean that there is more hemicellulose to interact with cellulose, resulting in improved water capture capacity [61]. The value ranges observed in these three properties were higher than, or similar to, those mentioned for other food co-products [27,58]. Due to their hydration properties, both ABSF and POSF, particularly at the smallest particle size, show the potential for use as ingredients in products that require hydration to improve yield or modify texture and viscosity, such as meat products, in which this characteristic promotes the formation of more strength gel in myofibrillar proteins.

#### 3.5.2. Oil Holding, Emulsifying, and Gelation Capacity

In the case of OHC (Table 3), this parameter was higher (*p* < 0.05) in POSF (values from 5.09 to 6.05 g/g) than in ABSF (values from 3.79 to 4.78 g/g). Regarding the sample size effect, the SI and the S sizes of both ABSF and POSF stand out for this property. Both flours showed higher OHC than other food co-products such as passionfruit (2.03 g/g), pineapple (1.57–1.85 g/g), and persimmon (2.15 g/g) [27]. In agreement with what was suggested above regarding the hydration properties, the elevated OHC obtained in the smallest sample sizes could be due to a high cellulose and low hemicellulose content. Furthermore, the observed increase in oil retention with decreasing sample size could be due to a higher contact surface area between oil and mushroom dietary fibre, since this contact surface would enhance the molecular interaction and van der Waals forces between oil particles and non-polar groups of dietary fibre [61]. The results of OHC should be taken into account before using ABSF and POSF as ingredients in fried products, as they could elicit a greasy sensation [62]. According to the results shown in Table 3, all emulsifying and gelation properties (EA and LGC) were discretely influenced by sample size in ABSF (*p* < 0.05), while in POSF, only LGC was influenced by granulometric factors (*p* < 0.05). For EA, ABSF exhibited a higher (*p* < 0.05) capacity to form emulsion than POSF (values from 11.67 to 23.21 and 3.48 to 3.54 mL/mL, respectively). This is likely due to the correlation between this property and protein content, which has been reported in previous works and calculated for the flours subject to this study (r = 0.99) [62]. Gelation capacity was expressed as the least gelation concentration (LGC); a low LGC means better gelation properties. The lowest LGC (*p* < 0.05) was obtained in ABSF-S and POSF-S (14.07 g/mL and 13.52 g/mL, respectively). Proteins can adsorb strongly at the oil–water interface through electrostatic and/or steric repulsive forces. This fact is influenced by the isoelectric point and interaction with other matrix compounds, such as polysaccharides [63]. Thus, differences in emulsifying and gelation properties could be associated with the interactions between, and relative ratio of, different mushroom constituents. The gelation properties observed for ABSF-S and POSF-S could be explained by the fact that there is more surface area contact for the interactions between water, oil, and functional groups [62]. Both flours, at different sample sizes, had LGC values similar to chickpeas, but only ABSF showed values ranging in EA similar to the results obtained for this legume [62]. According to the emulsifying properties analysed, both mushroom flours, but especially ABSF, could be used as emulsifying or stabilizing agents in foods like ice creams, chocolate, butter, or meat products.

### 3.6. Amino Acids Profile Analysis

**Figure 3:**
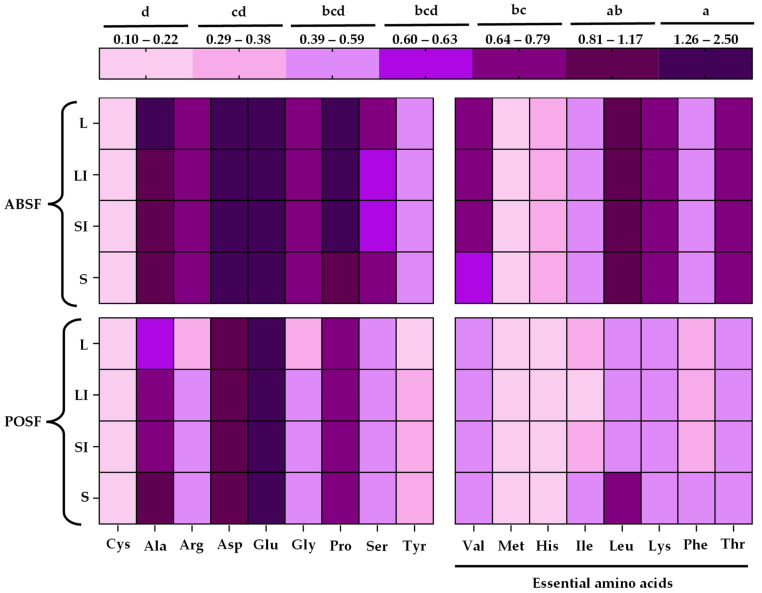
Effect of different sample sizes on amino acids profile of *A. bisporus* stems flour (ABSF) and *P. ostreatus* stems flour (POSF). Results are reported as mean ± SD (n = 3). Mean values within interval colors followed by different superscript letters (a–d) are significantly different when subjected to Tukey’s test (*p* < 0.05). L: >0.510 mm; LI: 0.510–0.315 mm; SI: 0.315–0.180 mm; S: <0.180 mm. Cys: L-cystine; Ala: L-alanine; Arg: L-arginine; Asp: L-apartic acid; Glu: L-glutamic acid; Gly: L-glycine; Pro: L-proline; Ser: L-serine; Tyr: L-tyrosine; Val: L-valine; Met: L-methionine; His: L-histidine hydrochloride monohydrate; Ile: L-isoleucine; Leu: L-leucine; Lys: L-lysine hydrochloride; Phe: L-phenylalanine; Thr: L-threonine. Results are expressed as g/100 g of flour.

In order to understand the qualities of the proteins previously analyzed, the amino acid profiles for each stems flour were examined, and the results are shown in Figure 3. The amount of amino acids was clearly influenced by species (*p* < 0.05), but only some of these amino acids were influenced by sample size (*p* < 0.05). To our knowledge, no studies have been published on the amino acid profile of PO stems; as a result, fruiting bodies were considered as a comparison point. The results obtained showed the same values (*p* > 0.05) for Cys, Met, and Glu in all species and sample sizes. Cys content in both POSF and ABSF (values between 0.10 and 0.22 g/100 g) was in consonance with that previously reported for PO, but higher than previously described for AB stems. Similar results were observed for the Met and Glu amount found in both species [3,64]. With regards to Ala, ABSF-L (1.28 g/100 g) showed a higher content (*p* < 0.05) than all sample sizes for POSF. Only the largest and intermediate sample sizes for ABSF exhibited a greater content of the amino acid Pro than POSF in all granulometries (*p* < 0.05). For both the Ala and Pro amino acids, ABSF and POSF results were in accordance with those previously reported [3,64]. There was no significant difference (*p* > 0.05) between species and sample size in the rest of the amino acids determined, and the results obtained were higher than those previously described for AB stems [3]. In the case of POSF, the values for the remaining amino acids were in agreement with previously reported in all cases except Ser (0.39–0.59 g/100 g) and Leu (0.39–0.67 g/100 g), in which the results obtained in this study were lower than the 0.91 and 1.01 g/100 g, respectively, described by Dabbour et al. [64]. Differences in amino acid content could be due to strain differences [65]. It is important to note that the samples comprised all essential amino acids; this high protein quality could make these flours a suitable ingredient for the enrichment of plant-based foods. In addition, some amino acids have been associated with flavours, such as umami flavour (Asp and Glu), so in this context, POSF and ABSF could have a high umami flavour based on the results of this work [66]. Because of this taste profile, ABSF and POSF could be incorporated into foods as salt substitutes.

### 3.7. Minerals Profile Analysis

Table 4 shows the data obtained in the minerals analysis. According to these results, both sample size and species influenced the mineral profile (*p* < 0.05). The most abundant element in both ABSF and POSF was potassium, followed by phosphorus (*p* < 0.05). A similar distribution has been observed previously in both mushroom stems [21,67]. ABSF was in general richer in minerals than POSF (*p* < 0.05), except in the case of zinc, for which POSF-S showed the highest amount (4.25 mg/100 g). The greatest difference (*p* < 0.05) in minerals between these species was observed for calcium (values from 428.23 to 700.77 and 1.25 to 21.56 mg/100 g flour, respectively). The highest calcium content was observed in ABSF-S (700.77 mg/100 g), which provides 88% of calcium’s nutrient reference values; thus, it might be considered for use as a potential source of calcium in the food industry [49]. In regards to sodium content, there was a positive correlation between this mineral and the sample size of ABSF (r = 0.99). The World Health Organization recommends a reduction in sodium intake to reduce blood pressure and risk of cardiovascular disease, stroke, and coronary heart disease in adults [68]. Taking into account the sodium content of the flours (Table 4), all sample sizes from POSF and ABSF-S could be suitable as potential ingredients for developing new functional foods low in sodium. Another important micronutrient for health is iron. Iron was more abundant in ABSF than in POSF (*p* < 0.05), and the highest value was observed in ABSF-S (39.61 mg/100 g). For all minerals, but especially iron, content levels are not typically as important as bioavailability is. According to Raman et al. [69], about 90% of the iron in edible mushrooms is easily absorbable. The mineral profile of PO and AB stems and fruiting bodies have been defined previously, and the range of values varied as a function of maturation stage, strain, location, and substrate. Therefore, the difference in the mineral profile between the flours subject to this study and those previously reported could be due to these factors [20,21,67,70,71].

### 3.8. Sugars and Organic Acids Profile Analysis

The sugar and organic acid profiles of the flours were discretely influenced by sample size and species (*p* < 0.05). According to the results (Table 5), sorbitol, sucrose, glucose, and trehalose were abundant sugars in the studied flours. Sorbitol was the predominant sugar in ABSF (*p* < 0.05), especially in the largest (26.60 mg/100 g) and large-intermediate (26.46 mg/100 g) sizes. Some authors reported mannitol as the most abundant sugar in the fruiting bodies of AB; this disagreement with our obtained results could be due to the morphological differences between fruiting bodies and stems, or due to mannitol metabolism, which promotes fructose, glucose, and sorbitol formation via glycolysis and galactose pathways [72,73]. Sucrose and glucose were the main sugars in POSF (*p* < 0.05). These results were in consonance with those previously reported for stems and fruiting bodies of PO [47,74]. Sucrose was found only in POSF, predominating in the smallest (6.59 mg/100 g) and small-intermediate (6.56 mg/100 g) sample sizes. The absence of this sugar in AB was previously reported for fruiting bodies [75]. On the other hand, glucose was found in both ABSF and POSF; its concentration was higher in POSF (*p* < 0.05), especially in the smallest (5.51 mg/100 g) and small-intermediate sizes (5.58 mg/100 g). However, in previous studies, glucose was the predominant sugar, in similar amounts, in AB and PO stems [47]. Trehalose content was higher in ABSF than in POSF, contrary to what has been reported previously for AB and PO fruiting bodies [72]. The difference in glucose concentration could be related to trehalose content, since trehalose is composed of two glucose molecules connected by an α−1,1-glycosidic bond, and its synthesis is performed by trehalose synthase complex (trehalose-6-phosphate synthase and trehalose-6- phosphate phosphatase) [76,77]. Additionally, glucose could be related to sorbitol resulting from the glycolysis and galactose pathways [73]. Furthermore, reduction in sugar content depends on many factors, such as ripening stage, strain, location, substrate, and the drying method [50]. The sugar profile of the stem flours is important for the participation of sugars in the Maillard reaction, as well as for the texture and sweetness of the food in which these ingredients could be integrated [38]. Sorbitol is used as a sweetener in the food industry; in this context, ABSF could act as a sweetener in new food formulations [76].

Species and sample size had a clear significant influence (*p* < 0.05) on organic acid content (Table 5). To our knowledge, no studies have been published on the organic acid profile of AB stems; to this end, fruiting bodies were considered for comparative purposes. Tartaric acid and acetic acid were more abundant (*p* < 0.05) in POSF than in ABSF, while lactic acid and isobutyric acid predominated in ABSF. The highest concentration of lactic acid was observed in ABSF-SI (7.69 mg/100 g); these results were contrary to those previously reported for the same strain of AB, in which lactic acid was not detected. This organic acid could have been formed from fructose in mitochondrial metabolism [78]. Conversely, the presence of lactic acid in PO stems had been previously reported [79]. It should be noted that citric acid was only detected in ABSF, and the highest amount (*p* < 0.05) was observed in ABSF-LI (0.93 mg/100 g), which was higher than previously reported [75,78]. In opposition to the results of this work, the presence of citric acid in PO stems has been previously observed [79]. Other authors have suggested that organic acids could influence the antioxidant activity of mushroom extract [78]. The organic acid profile of both flours was within the range observed in other food co-products [26]. In this context, both flours could be a potential ingredient in the development of functional foods. Citric acid can also extend the shelf life of mushrooms and prevent browning, so ABSF could be a more stable flour [78].

### 3.9. Fatty Acids Profile

The fatty acid profiles (Table 6) for each type of stems flour were discretely influenced by sample size and species (*p* < 0.05). In previous works, both AB and PO fruiting bodies and stems showed higher percentages of polyunsaturated fatty acids (PUFA) than monounsaturated fatty acids (MUFA) [47,72]. In this study, MUFAs made up the dominant percentage of fatty acids in ABSF (values ranging between 60.66 and 63.48%), while in POSF PUFAs were more abundant, especially in the small-intermediate particle size (55.71%). This result was in agreement with the 54.06% previously reported for PO fruiting bodies [74]. It is known that mushrooms are a rich source of fatty acids such as palmitic and linoleic acid [50,70]. The highest concentration (*p* < 0.05) of palmitic acid was detected in POSF-LI (25.22 g/100 g), while in ABSF the values ranged from 12.16 to 14.53 g/100 g, which is higher than the previously reported for AB stems [47]. Results for linoleic acid in POSF were highest in POSF-SI (39.86 g/100 g), similar to those previously reported for PO stems [47]. C18:2 (n 6, 9) was not detected in ABSF, which could be due to enzymatic degradation in 1-octen-3-ol [80]. With regards to the oleic acid content, there was a higher concentration observed in ABSF (values between 59.50 and 62.76 g/100 g) than those previously reported for AB stems [47]. In the case of POSF, the amount of C18:1 was lower than in ABSF (*p* < 0.05). The highest content of this fatty acid in POSF samples was obtained in the large-intermediate sample (27.88 g/100 g), which was in concordance with previously reported results for PO stems [47]. Another polyunsaturated fatty acid detected only in POSF was C20:5. The other fatty acids represented minor fractions (<5%). Linoleic acid is an essential fatty acid due to its involvement in docosapentaenoic acid (ω6) biosynthesis; based on these results, POSF could be more suitable for producing functional food enrichment via omega 6 [50].

### 3.10. Antioxidant Compounds and Capacity

#### 3.10.1. Assessment of the Total Phenolic Content

**Figure 4:**
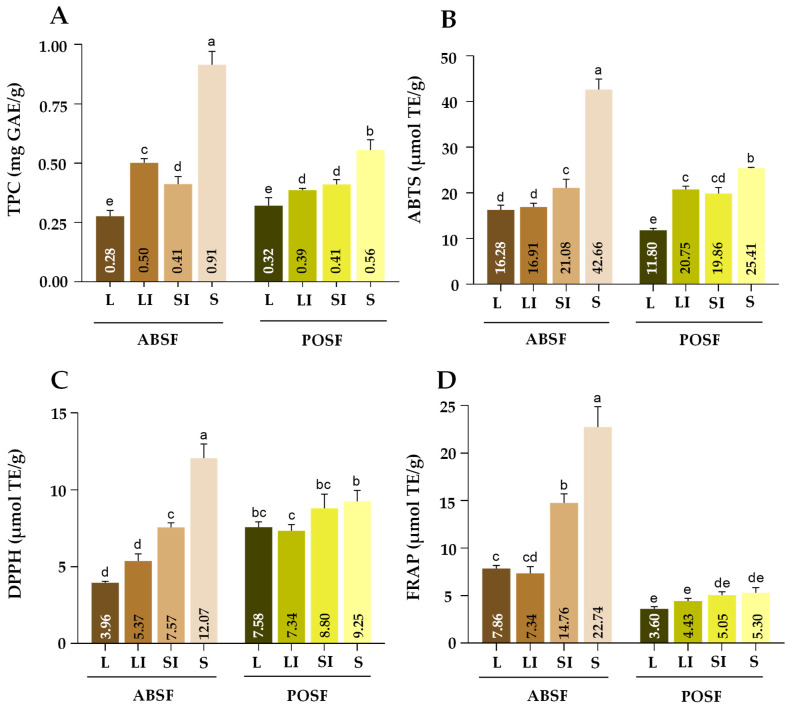
Effect of different sample sizes on TPC (**A**), ABTS (**B**), DPPH (**C**), and FRAP (**D**) of *A. bisporus* stems flour (ABSF) and *P. ostreatus* stems flour (POSF). Results are reported as mean ± SD (n = 3). Different letters (a–e) in the same graphic are significantly different when subjected to Tukey’s test (*p* < 0.05). L: >0.510 mm; LI: 0.510–0.315 mm; SI: 0.315–0.180 mm; S: <0.180 mm. TPC: total phenolic content; GAE: gallic acid equivalent; TE: Trolox equivalent.

The TPC of the flours analysed are shown in Figure 4A. According to the results obtained, sample size and species influenced the TPC evaluated by the Folin-Ciocalteau method (*p* < 0.05). The highest amount (0.91 mg/g) was obtained for ABSF-S (*p* < 0.05). This value was substantially lower than those previously reported for AB and PO stems in other studies, but similar to those observed for the same strain of AB (1.33 mg/g DW) [3,21,47,78]. POSF-S also showed the highest phenolic content (0.56 mg/g) among the POSF samples (*p* < 0.05). It is generally known that polyphenol oxidase (PPO) is a copper-containing oxidase correlated with phenolic content [81]. The much lower content of TPC in ABSF and POSF compared to that which has previously reported could be due to cultivation factors, or due to an increase in PPO activity derived from flour processing. During this procedure, the tissue could have been damaged, resulting in the consequent release of phenols into the intracellular space, where they could have been oxidized by PPO. Furthermore, the drying process at 50 °C does not inactivate this enzyme [82]. The results of this work indicated a negative correlation between TPC and sample size in both ABSF and POSF (r = −0.76 and r = −0.89, respectively). This distribution of TPC in the mushroom flours being dependent on sample size agrees with previous works on other food co-products [26]. This fact could be due to a higher extraction of compounds facilitated by a larger surface area in contact with the solvent. According to these results, the flours comprising the smallest sample sizes could have a higher amount of bioactive compounds such as phenolic compounds.

#### 3.10.2. Evaluation of the In Vitro Antioxidant Capacity

The TEAC values of ABTS, FRAP, and DPPH assay in both ABFS and POSF are shown in Figure 4. According to these results, the antioxidant activity of the flours under study was influenced by sample size and species (*p* < 0.05). The evaluation of antioxidant activity is a parameter of difficult comparison due to the different processing and extraction methods and manners of data reporting applied between studies [41]. In order to compare the results of the antioxidant assays of the studied flours with those previously reported for PO and AB fruiting bodies and stems, EC_50_ values were calculated by interpolation in the curve obtained in the methanolic extract of ABSF and POSF before TEAC. To our knowledge, no studies have been published on the TEAC and EC50 values of AB and PO stems, so fruiting bodies were considered for comparison.

The results for ABTS and DPPH are shown in Figure 4B,C. ABSF-S exhibited the highest values (*p* < 0.05) in both analyses (42.66 and 12.07 µmol TE/g), followed by POSF-S (25.41 and 9.25 µmol TE/g). With regards to the FRAP assay (Figure 4D), the highest (*p* < 0.05) reducing power of Fe^3+^ was again observed in ABSF-S. These TEAC values were higher than those reported by Smolskait et al. [83] in a methanolic extract from AB and PO fruiting bodies (DPPH• 0.13 and 0.74 µmol TE/g, respectively; ABTS^•+^ 1.41 and 0.65 µmol TE/g, respectively; FRAP 2.09 and 1.49 µmol TE/g, respectively). However, phenolic content in the flours was lower than those reported by Smolskait et al. [83], which could mean that the antioxidant activity from the mushrooms might be due to other bioactive compounds with reducing capacities present in the extract, such as β-glucans, citric acid, trehalose, and zinc [55,76,78,84]. The β-glucan content of the extract was calculated in order to correlate this compound with antioxidant activity, and there was a positive correlation between β-glucan content and ABTS, DPPH, and FRAP assays in POSF extracts (r = 0.93; r = 0.87; and r = 0.94 respectively).

For the DPPH assay, the EC_50_ from ABSF (values between 0.68 and 1.96 mg of flour/mL) was lower than that reported by Tajalli et al. [85] (5.19 mg/mL) and Gąsecka et al. [78] (3.2 mg/mL) for the same strain, but higher than that analysed by Öztürk et al. [86] (0.988 mg/mL) in all sample sizes except the finest. In the case of POSF, the EC50 from the DPPH assay (values between 1.82 and 2.24 mg/mL) were lower than previously indicated in the literature (8.4 mg/mL) [87]. Although the antioxidant activity shown in this co-product was lower than that reported in other co-products rich in polyphenols such as cacao shell, the antioxidant activity of these mushroom stems was higher than that described for some tropical fruit co-products (5.76 µmol TE/g in DPPH, 13.46 µmol TE/g in ABTS) and fig co-product [41,58,88]. According to the antioxidant activity profiles determined, the smallest sample sizes for both mushroom stem flours, but especially ABSF-S, could be a potential ingredient for the development of functional food. Although this methodology is a good tool to obtain an initial idea regarding the antioxidant activity of the flours, it is necessary to study this activity in vivo and with a previous digestion process to affirm this. Another thing to take into account is the interaction with the matrix of the food in which the flour would be incorporated.

## 4. Conclusions

Sample size and species influenced the characterization and antioxidant activity of both flours. ABSF was highlighted for its protein, calcium, and sorbitol content. Importantly, a reduction in sample size increased the TPC and antioxidant activity in vitro. There could be several food applications for this flour as an emulsifier, stabilizer, sweetener, or fortifier in the development of new foods, based mainly on its protein content and the presence of all of the essential amino acids. Additionally, ABSF-S could be a suitable and interesting ingredient in functional food improvement based on its antioxidant activity. On the other hand, POSF showed the highest dietary fibre and linoleic acid content. Based on this result, POSF might be used as a source of dietary fibre and omega-6 in the development of functional foods. It should be noted that POSF-S showed the highest value of β-glucans, a molecule correlated with antioxidant activity. Furthermore, the smallest sample size for both species also appears suitable for the production of food that requires hydration properties, and for the formulation of functional food with a low sodium content. Importantly, the smallest sample size of both species showed higher yield and, consequently, the obtention of these flours could be more economical. This strategy of valorization of mushroom co-products is part of the circular bioeconomy and can be implemented by local farmers, generating a new income opportunity for the primary sector. However, it is necessary to understand production costs, the bioaccessibility of all compounds analysed in the flours, and their interactions with the potential food matrix.

## Figures and Tables

**Table 1:** Effect of different sample sizes on proximate composition and D-glucan content of *A. bisporus* stems flour (ABSF) and *P. ostreatus* stems flour (POSF).

	ABSF				POSF			
	L	LI	SI	S	L	LI	SI	S
Moisture	5.01 ^d^ ± 0.10	4.74 ^d^ ± 0.13	4.84 ^d^ ± 0.06	5.43 ^c^ ± 0.17	8.05 ^b^ ± 0.16	8.05 ^b^ ± 0.18	8.46 ^ab^ ± 0.18	8.78 ^a^ ± 0.15
Protein	14.17 ^a^ ± 0.03	14.21 ^a^ ± 0.11	14.25 ^a^ ± 0.25	14.36 ^a^ ± 0.04	8.04 ^d^ ± 0.07	9.02 ^bc^ ± 0.06	8.91 ^c^ ± 0.10	9.42 ^b^ ± 0.03
Lipid	2.08 ^e^ ± 0.06	2.51 ^d^ ± 0.05	2.64 ^cd^ ± 0.07	3.61 ^a^ ± 0.08	2.22 ^e^ ± 0.10	2.59 ^d^ ± 0.09	2.93 ^b^ ± 0.02	2.82 ^bc^ ± 0.00
Ash	8.12 ^a^ ± 0.06	8.12 ^a^ ± 0.06	8.24 ^a^ ± 0.26	8.33 ^a^ ± 0.09	7.22 ^b^ ± 0.36	6.75 ^bc^ ± 0.05	6.75 ^bc^ ± 0.14	6.27 ^c^ ± 0.09
Total Carbohydrates	70.63 ^c^ ± 0.17	70.42 ^c^ ± 0.24	70.02 ^c^ ± 0.44	68.27 ^d^ ± 0.26	74.48 ^a^ ± 0.64	73.60 ^ab^ ± 0.16	72.94 ^b^ ± 0.19	72.70 ^b^ ± 0.27
IDF	46.94 ^cd^ ± 2.54	48.23 ^c^ ± 2.82	41.61 ^d^ ± 0.09	33.66 ^e^ ± 3.15	63.04 ^a^ ± 1.83	61.75 ^a^ ± 0.71	58.34 ^ab^ ± 0.99	54.78 ^b^ ± 0.10
SDF	1.41 ^c^ ± 0.65	1.65 ^c^ ± 0.83	2.39 ^bc^ ± 0.19	3.51 ^b^ ± 0.09	3.21 ^b^ ± 0.15	2.28 ^c^ ± 0.08	3.19 ^b^ ± 0.39	5.06 ^a^ ± 0.43
TDF	48.35 ^cd^ ± 1.89	49.89 ^c^ ± 2.15	44.00 ^d^ ± 0.22	37.17 ^e^ ± 3.24	66.25 ^a^ ± 1.79	64.07 ^ab^ ± 0.62	61.76 ^ab^ ± 0.91	59.84 ^b^ ± 2.50
α-glucans	8.48 ^f^ ± 0.23	9.46 ^e^ ± 0.51	8.97 ^ef^ ± 0.19	13.86 ^a^ ± 0.33	12.72 ^b^ ± 0.54	11.49 ^c^ ± 0.05	12.20 ^bc^ ± 0.23	10.45 ^d^ ± 0.09
β-glucans	13.11 ^c^ ± 0.52	13.41 ^c^ ± 0.34	13.85 ^c^ ± 0.19	9.51 ^d^ ± 0.24	36.62 ^b^ ± 2.69	39.00 ^ab^ ± 1.10	39.33 ^ab^ ± 1.79	40.34 ^a^ ± 0.60
D-glucans	21.59 ^b^ ± 0.35	22.88 ^b^ ± 0.61	22.87 ^b^ ± 0.05	22.86 ^b^ ± 1.18	49.34 ^a^ ± 2.15	50.50 ^a^ ± 1.07	51.52 ^a^ ± 1.58	50.79 ^a^ ± 0.55

Results are reported as mean ± SD (n = 3). Mean values within rows followed by different superscript letters (a–f) are significantly different when subjected to Tukey’s test (*p* < 0.05). L: >0.510 mm; LI: 0.510–0.315 mm; SI: 0.315–0.180 mm; S: <0.180 mm. IDF: insoluble dietary fibre, SDF: soluble dietary fibre, TDF: total dietary fibre. Results are expressed as g/100 g of flour.

**Table 2:** Effect of different sample sizes on physicochemical characteristics of *A. bisporus* stems flour (ABSF) and *P. ostreatus* stems flour (POSF).

	Sample Size	pH	Aw	Colour					
				L*	a*	b*	C*	h*	ΔE*
ABSF	L	6.16 ^a^ ± 0.01	0.38 ^h^ ± 0.00	64.60 ^e^ ± 1.96	4.51 ^a^ ± 0.38	14.12 ^a^ ± 0.51	14.82 ^c^ ± 0.59	72.29 ^e^ ± 0.95	-
	LI	6.21 ^a^ ± 0.04	0.39 ^g^ ± 0.00	65.02 ^e^ ± 1.70	4.76 ^a^ ± 0.22	14.79 ^a^ ± 0.21	15.54 ^c^ ± 0.22	72.15 ^de^ ± 0.77	0.90 ^d^ ± 0.02
	SI	6.18 ^a^ ± 0.01	0.41 ^f^ ± 0.00	68.64 ^d^ ± 0.43	4.42 ^a^ ± 0.11	15.06 ^a^ ± 0.20	15.69 ^c^ ± 0.22	73.66 ^de^ ± 0.27	4.04 ^c^ ± 0.43
	S	6.18 ^a^ ± 0.11	0.42 ^e^ ± 0.00	75.01 ^c^ ± 0.39	3.99 ^a^ ± 0.13	14.73 ^a^ ± 0.29	15.26 ^c^ ± 0.31	74.83 ^d^ ± 0.22	10.42 ^a^ ± 0.40
POSF	L	6.15 ^a^ ± 0.06	0.44 ^d^ ± 0.00	75.14 ^c^ ± 1.34	4.24 ^a^ ± 0.44	28.45 ^b^ ± 1.38	29.27 ^ab^ ± 1.65	81.66 ^c^ ± 0.89	-
	LI	6.15 ^a^ ± 0.05	0.46 ^c^ ± 0.00	82.15 ^b^ ± 0.83	1.39 ^b^ ± 0.46	27.68 ^b^ ± 1.02	27.71 ^b^ ± 1.03	87.12 ^b^ ± 0.93	6.80 ^b^ ± 0.40
	SI	6.12 ^a^ ± 0.04	0.49 ^b^ ± 0.00	80.97 ^b^ ± 0.48	1.98 ^b^ ± 0.75	29.62 ^b^ ± 1.07	29.69 ^a^ ± 1.11	86.20 ^b^ ± 1.35	6.08 ^b^ ± 0.50
	S	6.07 ^a^ ± 0.09	0.50 ^a^ ± 0.00	84.88 ^a^ ± 0.49	0.18 ^c^ ± 0.06	29.37 ^b^ ± 0.57	29.37 ^ab^ ± 0.57	89.65 ^a^ ± 0.10	10.45 ^a^ ± 0.80

Results are reported as mean ± SD (n = 3). Mean values within rows followed by different superscript letters (a–h) are significantly different when subjected to Tukey’s test (*p* < 0.05). L: >0.510 mm; LI: 0.510–0.315 mm; SI: 0.315–0.180 mm; S: <0.180 mm. Aw: Water activity. L*: Lightness, a*: redness, b*: yellowness, C*: chroma, h*: hue, ΔE*: color difference.

**Table 3:** Effect of different sample sizes on techno-functional properties of *A. bisporus* stems flour (ABSF) and *P. ostreatus* stems flour (POSF).

	Sample Size	WHC (g/g)	OHC (g/g)	WAC (g/g)	EA (mL/mL)	SWC (mL/g)	LGC (g/mL)
ABSF	L	5.70 ^b^ ± 0.38	3.79 ^d^ ± 0.08	3.66 ^bc^ ± 0.09	11.67 ^b^ ± 0.90	8.05 ^c^ ± 0.41	20.45 ^a^ ± 0.82
	LI	6.84 ^a^ ± 0.60	3.61 ^d^ ± 0.42	3.79 ^bc^ ± 0.16	22.08 ^a^ ± 0.98	12.13 ^b^ ± 0.64	17.19 ^b^ ± 1.19
	SI	5.17 ^b^ ± 0.35	4.23 ^cd^ ± 0.48	4.36 ^b^ ± 0.04	23.21 ^a^ ± 1.79	9.50 ^c^ ± 0.50	15.91 ^bc^ ± 0.66
	S	5.03 ^b^ ± 0.06	4.78 ^c^ ± 0.22	5.27 ^a^ ± 0.06	13.69 ^b^ ± 1.03	15.39 ^a^ ± 0.23	14.07 ^c^ ± 0.01
POSF	L	3.81 ^c^ ± 0.19	5.09 ^bc^ ± 0.16	3.01 ^c^ ± 0.45	3.54 ^c^ ± 0.04	12.55 ^b^ ± 0.66	19.52 ^a^ ± 1.10
	LI	3.64 ^c^ ± 0.48	5.58 ^ab^ ± 0.17	2.93 ^c^ ± 0.14	3.52 ^c^ ± 0.02	15.19 ^a^ ± 1.03	16.68 ^bc^ ± 1.16
	SI	3.92 ^c^ ± 0.43	6.05 ^a^ ± 0.07	3.51 ^c^ ± 0.44	3.48 ^c^ ± 0.07	16.06 ^a^ ± 0.41	15.04 ^bc^ ± 1.07
	S	3.93 ^c^ ± 0.30	5.99 ^a^ ± 0.13	4.10 ^b^ ± 0.34	3.51 ^c^ ± 0.07	16.03 ^a^ ± 0.54	13.52 ^c^ ± 1.24

Results are reported as mean ± SD (n = 3). Mean values within rows followed by different superscript letters (a–d) are significantly different when subjected to Tukey’s test (*p* < 0.05). L: >0.510 mm; LI: 0.510–0.315 mm; SI: 0.315–0.180 mm; S: <0.180 mm. WHC: water holding capacity, OHC: oil holding capacity, WAC: water absorption capacity, EA: emulsifying activity, SWC: swelling capacity, LGC: least gelation concentration.

**Table 4:** Effect of different sample sizes on minerals profile of *A. bisporus* stems flour (ABSF) and *P. ostreatus* stems flour (POSF).

	Sample Size	Ca	Cu	Fe	K	Mg	Mn	Na	P	Zn
ABSF	L	428.23 ^d^ ± 15.72	1.80 ^c^ ± 0.01	18.63 ^d^ ± 0.95	2422.86 ^a^ ± 35.19	150.43 ^b^ ± 1.85	0.66 ^d^ ± 0.01	201.12 ^a^ ± 1.39	1851.42 ^c^ ± 8.43	3.12 ^e^ ± 0.05
	LI	504.79 ^c^ ± 5.48	1.99 ^a^ ± 0.02	22.81 ^c^ ± 1.14	2234.12 ^bc^ ± 13.63	153.83 ^ab^ ± 3.15	0.86 ^c^ ± 0.00	193.88 ^ab^ ± 9.80	1833.66 ^c^ ± 16.58	3.56 ^d^ ± 0.03
	SI	545.62 ^b^ ± 26.00	2.04 ^a^ ± 0.02	27.53 ^b^ ± 1.47	2163.76 ^c^ ± 13.84	159.76 ^a^ ± 1.59	0.97 ^b^ ± 0.01	183.86 ^b^ ± 0.10	1906.28 ^b^ ± 8.81	3.76 ^c^ ± 0.01
	S	700.77 ^a^ ± 18.88	2.00 ^a^ ± 0.03	39.61 ^a^ ± 0.67	2157.18 ^c^ ± 30.59	157.61 ^a^ ± 3.28	1.19 ^a^ ± 0.02	170.10 ^c^ ± 5.59	1964.96 ^a^ ± 10.49	3.95 ^b^ ± 0.04
POSF	L	1.25 ^e^ ± 0.02	1.17 ^e^ ± 0.02	4.47 ^e^ ± 0.08	2164.94 ^c^ ± 34.13	66.04 ^c^ ± 1.37	0.38 ^g^ ± 0.00	62.43 ^e^ ± 1.35	1077.52 ^de^ ± 2.37	2.30 ^h^ ± 0.02
	LI	7.47 ^e^ ± 0.38	1.42 ^d^ ± 0.01	4.61 ^e^ ± 0.05	2375.66 ^a^ ± 45.69	52.94 ^e^ ± 1.91	0.44 ^f^ ± 0.01	79.51 ^d^ ± 1.18	1067.37 ^e^ ± 14.12	2.71 ^g^ ± 0.02
	SI	17.41 ^e^ ± 0.59	1.37 ^d^ ± 0.03	4.69 ^e^ ± 0.27	2211.53 ^bc^ ± 3.84	52.48 ^e^ ± 1.42	0.43 ^f^ ± 0.00	77.41 ^d^ ± 1.43	1000.64 ^f^ ± 10.38	2.89 ^f^ ± 0.02
	S	21.56 ^e^ ± 0.38	1.87 ^b^ ± 0.02	6.68 ^e^ ± 0.13	2290.44 ^b^ ± 22.89	59.39 ^d^ ± 0.83	0.48 ^e^ ± 0.02	75.31 ^d^ ± 0.71	1100.92 ^d^ ± 5.44	4.25 ^a^ ± 0.04

Results are reported as mean ± SD (n = 3). Mean values within rows followed by different superscript letters (a–h) are significantly different when subjected to Tukey’s test (*p* < 0.05). L: >0.510 mm; LI: 0.510–0.315 mm; SI: 0.315–0.180 mm; S: <0.180 mm. Ca: calcium, Cu: copper, Fe: iron, K: potassium, Mg: magnesium, Mn: manganese, Na: sodium, P: phosphorus, Zn: zinc. Results are expressed as mg/100 g of flour.

**Table 5:** Effect of different sample sizes on sugar and organic acid profiles of *A. bisporus* stems flour (ABSF) and *P. ostreatus* stems flour (POSF).

	Compounds	ABSF				POSF			
		L	LI	SI	S	L	LI	SI	S
Sugars	Maltitol	0.56 ^b^ ± 0.03	0.47 ^b^ ± 0.01	0.81 ^a^ ± 0.05	0.82 ^a^ ± 0.03	n.d.	n.d.	n.d.	n.d.
	Sucrose	n.d.	n.d.	n.d.	n.d.	4.24 ^c^ ± 0.09	5.76 ^b^ ± 0.21	6.56 ^a^ ± 0.08	6.59 ^a^ ± 0.19
	Glucose	0.56 ^d^ ± 0.01	0.50 ^d^ ± 0.01	0.56 ^d^ ± 0.01	0.54 ^d^ ± 0.00	3.63 ^c^ ± 0.04	5.02 ^b^ ± 0.26	5.85 ^a^ ± 0.10	5.51 ^a^ ± 0.36
	Sorbitol	26.60 ^a^ ± 0.33	26.46 ^a^ ± 0.31	24.78 ^ab^ ± 0.82	22.57 ^b^ ± 2.10	1.12 ^c^ ± 0.19	2.61 ^c^ ± 0.15	1.81 ^c^ ± 0.06	2.28 ^c^ ± 0.36
	Trehalose	5.53 ^b^ ± 0.20	6.11 ^a^ ± 0.06	6.53 ^a^ ± 0.29	4.72 ^c^ ± 0.08	1.79 ^d^ ± 0.03	1.83 ^d^ ± 0.05	1.87 ^d^ ± 0.06	1.72 ^d^ ± 0.26
Organic Acids	Tartaric acid	0.29 ^e^ ± 0.00	0.29 ^e^ ± 0.04	0.22 ^e^ ± 0.02	0.30 ^e^ ± 0.04	1.95 ^d^ ± 0.08	2.42 ^c^ ± 0.10	2.58 ^b^ ± 0.07	2.79 ^a^ ± 0.01
	Lactic acid	6.40 ^b^ ± 0.05	6.88 ^b^ ± 0.22	7.69 ^a^ ± 0.40	6.22 ^b^ ± 0.28	2.09 ^c^ ± 0.01	2.15 ^c^ ± 0.01	2.11 ^c^ ± 0.05	2.23 ^c^ ± 0.13
	Acetic acid	2.45 ^b^ ± 0.13	3.03 ^b^ ± 0.22	2.61 ^b^ ± 0.11	2.93 ^b^ ± 0.04	34.74 ^a^ ± 0.07	34.90 ^a^ ± 0.52	34.49 ^a^ ± 0.59	34.64 ^a^ ± 0.62
	Isobutyric acid	21.44 ^a^ ± 0.30	17.89 ^b^ ± 1.55	17.19 ^b^ ± 0.33	16.38 ^c^ ± 1.25	11.39 ^d^ ± 0.05	11.39 ^d^ ± 0.53	11.22 ^d^ ± 0.04	11.87 ^d^ ± 0.38
	Citric acid	0.78 ^b^ ± 0.01	0.93 ^a^ ± 0.05	0.86 ^ab^ ± 0.01	0.84 ^b^ ± 0.03	n.d.	n.d.	n.d.	n.d.

Results are reported as mean ± SD (n = 3). Mean values within rows followed by different superscript letters (a–e) are significantly different when subjected to Tukey’s test (*p* < 0.05). L: >0.510 mm; LI: 0.510–0.315 mm; SI: 0.315–0.180 mm; S: <0.180 mm; n.d.: not detected. Results are expressed as mg/100 g of flour.

**Table 6:** Effect of different sample sizes on fatty acids profile of *A. bisporus* stems flour (ABSF) and *P. ostreatus* stems flour (POSF).

Compounds	ABSF				POSF			
	L	LI	SI	S	L	LI	SI	S
C4:0	n.d.	n.d.	n.d.	n.d.	0.49 ^d^ ± 0.01	0.74 ^b^ ± 0.01	0.92 ^a^ ± 0.01	0.55 ^c^ ± 0.01
C10:0	n.d.	n.d.	n.d.	n.d.	1.04 ^b^ ± 0.01	1.04 ^b^ ± 0.01	2.39 ^a^ ± 0.01	0.98 ^c^ ± 0.01
C13:0	n.d.	n.d.	n.d.	n.d.	0.78 ^d^ ± 0.01	0.94 ^c^ ± 0.01	2.32 ^a^ ± 0.01	1.15 ^b^ ± 0.01
C14:0	0.66 ^c^ ± 0.02	0.69 ^c^ ± 0.04	0.64 ^c^ ± 0.05	0.96 ^b^ ± 0.01	5.52 ^a^ ± 0.01	n.d.	n.d.	n.d.
C15:0	1.31 ^a^ ± 0.14	0.88 ^b^ ± 0.02	1.21 ^a^ ± 0.01	1.36 ^a^ ± 0.01	0.68 ^c^ ± 0.01	0.66 ^c^ ± 0.01	1.37 ^a^ ± 0.01	0.82 ^bc^ ± 0.02
C16:0	14.53 ^d^ ± 0.20	12.16 ^f^ ± 0.07	14.43 ^d^ ± 0.08	13.56 ^e^ ± 0.06	19.28 ^b^ ± 0.01	25.22 ^a^ ± 0.03	11.17 ^f^ ± 0.02	17.96 ^c^ ± 0.01
C16:1	0.73 ^f^ ± 0.06	0.72 ^f^ ± 0.02	1.08 ^d^ ± 0.02	0.95 ^e^ ± 0.01	1.17 ^c^ ± 0.01	1.14 ^cd^ ± 0.01	2.19 ^a^ ± 0.01	1.45 ^b^ ± 0.02
C18:0	5.84 ^c^ ± 0.38	5.71 ^cd^ ± 0.04	5.88 ^c^ ± 0.02	5.45 ^d^ ± 0.02	4.51 ^e^ ± 0.01	9.72 ^a^ ± 0.03	1.64 ^f^ ± 0.01	7.05 ^b^ ± 0.02
C18:1	59.93 ^c^ ± 0.28	62.76 ^a^ ± 0.11	61.21 ^b^ ± 0.91	59.50 ^c^ ± 0.16	16.48 ^f^ ± 0.01	27.88 ^d^ ± 0.01	10.75 ^g^ ± 0.01	20.27 ^e^ ± 0.01
C18:2 (n 6, 9)	n.d.	n.d.	n.d.	n.d.	23.95 ^c^ ± 0.03	20.57 ^d^ ± 0.03	39.86 ^a^ ± 0.01	35.06 ^b^ ± 0.04
C20:1	n.d.	n.d.	n.d.	n.d.	0.98 ^d^ ± 0.01	1.30 ^c^ ± 0.01	3.03 ^a^ ± 0.01	1.75 ^b^ ± 0.02
C20:5 (n 5, 8, 11, 14, 17)	n.d.	n.d.	n.d.	n.d.	23.24 ^a^ ± 0.03	7.02 ^d^ ± 0.04	15.85 ^b^ ± 0.02	7.62 ^c^ ± 0.02
Total SFA (% of total FA)	22.33 ^d^ ± 0.40	19.44 ^f^ ±0.15	22.16 ^d^ ± 0.10	21.33 ^e^ ± 0.07	26.50 ^c^ ± 0.03	38.31 ^a^ ± 0.02	19.80 ^f^ ± 0.06	28.50 ^b^ ± 0.08
Total MUFA (% of total FA)	60.66 ^c^ ± 0.23	63.48 ^a^ ±0.09	62.29 ^b^ ± 0.93	60.46 ^c^ ± 0.16	18.63 ^f^ ± 0.02	30.32 ^d^ ± 0.02	15.98 ^g^ ± 0.02	23.47 ^e^ ± 0.04
Total PUFA (% of total FA)	n.d.	n.d.	n.d.	n.d.	47.19 ^b^ ± 0.06	27.59 ^d^ ± 0.04	55.71 ^a^ ± 0.01	42.68 ^c^ ± 0.04

Results are reported as mean ± SD (n = 3). Mean values within rows followed by different superscript letters (a–g) are significantly different when subjected to Tukey’s test (*p* < 0.05). L: >0.510 mm; LI: 0.510–0.315 mm; SI: 0.315–0.180 mm; S: <0.180 mm; n.d.: non detected. FA: fatty acid; SFA: saturated fatty acid; MUFA: monounsaturated fatty acid; PUFA: polyunsaturated fatty acid. Results are expressed as a percentage (%) of total fatty acids.

## Data Availability

Data are contained within the article or Supplementary Materials.

## Supplementary Materials

The following supporting information can be downloaded at: https://www.mdpi.com/article/10.3390/antiox13030349/s1, Table S1: Glucans composition of extracts of *Agaricus bisporus* stems flour (ABSF), and *Pleurotus ostreatus* stems flour (POSF).
