# Supplementary material for: Evaluation of Sample Size Influence on Chemical Characterization and In Vitro Antioxidant Properties of Flours Obtained from Mushroom Stems Coproducts
Source: Antioxidants (Basel). 2024 Mar 14;13(3):349. doi: 10.3390/antiox13030349 (PMC10968205; doi:10.3390/antiox13030349)
Supplement: Supplementary file 1 [file antioxidants-13-00349-s001.zip › antioxidants-2896162-supplementary.pdf]

Development and characterization of a value-added product suitable for food applications from *Agaricus bisporus* and *Pleurotus ostreatus* stems: Evaluation of particle size influence and *in vitro* antioxidant activity

Patricia Bermúdez-Gómez[1], J. Fernández-López[2], Margarita Pérez-Clavijo[1], M. Viuda-Martos[2]

1 Centro Tecnológico de Investigación del Champiñón en La Rioja (CTICH), Carretera Calahorra, KM 4, 26560 Autol, La Rioja, Spain.

2 IPOA Research Group, Agro-Food Technology Department, Escuela Politécnica Superior de Orihuela, Crta. Beniel km 3.2, Miguel Hernández University, 03300 Orihuela, Alicante, Spain

Table S1. Glucans composition of extracts of *Agaricus bisporus* stems flour (ABSF), and *Pleurotus ostreatus* stems flour (POSF).

Sample	Particle size (mm)	D-glucans	α-glucans	β-glucans
ABSF extracts	L	11.53 ± 0.14^{e}	8.63 ± 0.21^{c}	2.90 ± 0.35^{f}
	LI	11.51 ± 0.73^{e}	5.70 ± 0.23^{d}	5.81 ± 0.51^{de}
	SI	8.92 ± 0.22^{f}	4.15 ± 0.06^{d}	4.77 ± 0.28^{ef}
	S	8.76 ± 0.05^{f}	4.70 ± 0.06^{d}	4.05 ± 0.04^{ef}
POSF extracts	L	22.54 ± 1.94^{d}	14.45 ± 1.55^{b}	8.09 ± 0.69^{d}
	LI	30.24 ± 0.89^{c}	15.32 ± 0.94^{a}	14.92 ± 1.59^{c}
	SI	36.63 ± 0.19^{b}	16.95 ± 1.00^{a}	19.68 ± 0.82^{b}
	S	41.33 ± 0.38^{a}	12.52 ± 1.17^{b}	28.81 ± 1.53^{a}

Results are reported as mean ± SD (n = 3). Mean values within rows followed by different superscript letters (a-f) are significantly different when subjected to Tukey's test ($p < 0.05$). L — >0.510 mm; LI 274 — 0.510-0.315 mm; SI—0.315-0.180 mm; S —<0.180 mm. Values are expressed as g/100 g of extract.
